# Why Wait? Early Determinants of School Dropout in Preventive Pediatric Primary Care

**DOI:** 10.1371/journal.pone.0142315

**Published:** 2015-11-10

**Authors:** Marie-José Theunissen, Hans Bosma, Petra Verdonk, Frans Feron

**Affiliations:** 1 Department of Social Medicine, School for Public Health and Primary Care (CAPHRI), Faculty of Health, Medicine and Life Sciences, Maastricht University, Maastricht, The Netherlands; 2 Youth Health Care division, Regional Public Health Service GGD Brabant-Zuidoost, Eindhoven, The Netherlands; 3 Department of Medical Humanities, EMGO Institute for Health and Care Research, School of Medical Sciences, VU University Medical Center, Amsterdam, The Netherlands; University of Perugia, ITALY

## Abstract

**Background:**

To answer the question of what bio-psychosocial determinants in infancy, early and middle childhood, and adolescence predict school drop-out in young adulthood, we approached the complex process towards school dropout as a multidimensional, life-course phenomenon. The aim is to find signs of heightened risks of school dropout as early as possible which will eventually help public health workers in reducing these risks.

**Methods:**

In a case-control design, we used data from both the Preventive Pediatric Primary Care (PPPC) files (that contain information from birth onwards) and additional questionnaires filled out by 529 youngsters, aged 18–23 years, and living in the South-east of the Netherlands. We first conducted univariate logistic regression analyses with school-dropout as the dependent variable. Backward and forward stepwise analyses with the significant variables were done with variables pertaining to the 0 to 4 year period. Remaining significant variables were forced into the next model and subsequently variables pertaining to respectively the 4 to 8, 8 to 12 and 12 to 16 year period were introduced in a stepwise analysis. All analyses were cross-validated in an exploratory and confirmatory random half of the sample.

**Results:**

One parent families and families with a non-Western background less often attended the health examinations of the PPPC and such less attendance was related to school dropout. The birth of a sibling (OR 0.63, 95% CI 0.43–0.93) in infancy and self-efficacy (OR 0.53, 95% CI 0.38–0.74) in adolescence decreased the odds of school dropout; externalizing behavior (OR 2.81, 95% CI 1.53–5.14) in middle childhood and (sickness) absence (OR 5.62, 95% CI 2.18–14.52) in adolescence increased the risks.

**Conclusion:**

To prevent school dropout, PPPC professionals should not wait until imminent dropout, but should identify and tackle risk factors as early as possible and actively approach youngsters who withdraw from public health care.

## Introduction

School dropout is an important but complex public health problem [1] that, when trying to tackle the problem, requires an integrative perspective and approach [2]. It is strongly associated with poor health and has major adverse socioeconomic consequences on both the macro- and personal level [3]. Dropout is not a discrete event, but a dynamic developmental process that often begins early in life [4,5]. Nevertheless, most interventions to prevent school dropout are school-based and targeted at factors that operate late in the dropout process [6,7]. Few interventions are aimed at tackling the problems at its roots by identifying children with physical, mental, psychosocial, or socioeconomic problems early in life. The aim of this study is to offer insight into the bio-psychosocial determinants of school dropout in infancy, early and middle childhood, and adolescence. This will contribute to develop more effective interventions in public health care aimed at reducing school dropout at an as early as possible stage.

Many factors that influence the process of early school leaving are already identified [8] and can be divided in student-related (e.g. overall poor health in childhood and adolescence [3], externalizing and internalizing behavior [9,10], life-style, personality [3,11,12], sex differences and gender beliefs [13], family-related (e.g. living in a single-parent family[14]) and school-related factors (e.g. little concern in school policies [15]). However, most studies until now are restricted to single determinants. Therefore, in the current study among Dutch young adolescents, we used a multidimensional approach with information from both the Preventive Pediatric Primary Care (PPPC) and an additional questionnaire. In The Netherlands, the PPPC offers longitudinal anticipatory guidance to all children between birth and 19 years [16,17] enabling us to perceive the determinants of school dropout from a life-course perspective.

The aim of this study is to find determinants of increased risks of school dropout as early as possible which will eventually help public health workers in reducing these risks. We used case-control data from the PPPC files and additional questionnaires from 529 Dutch youngsters to examine which bio-psychosocial determinants in infancy, childhood and adolescence predict school drop-out in young adulthood (18–23 years). An age-attentive stepwise modeling allowed for the identification of determinants as early in life as possible. This study has an explorative character and cross-validating the analyses in a random exploratory and confirmatory half of the sample minimized the possibility of capitalization on chance.

## Methods

### Study population

This study is part of the Dutch SIODO (Stay In Or Drop Out) study, a sequential mixed-methods study focusing on the early identification of risk factors on the pathway to school dropout [18]. The current paper is based on the quantitative case-control data from SIODO in which school dropouts are compared with a control group with regard to medical, developmental, environmental, psychosocial and personality- and lifestyle- related determinants in infancy, childhood and adolescence. In November 2011, the municipal compulsory education department of the city of Eindhoven, The Netherlands selected all young adults aged 18 to 23 years who did not yet attain a basic qualification on August 1st, 2010, but who all still attended school at the start of that school year. This minimum qualification (for making a good entry on the labor market) is equivalent to higher general secondary education, pre-university education or intermediate vocational education, Level 2 [19,20]. At this age, generally young adults have obtained this required qualification. We excluded cognitively impaired young people (IQ < 70) and young people exempt from compulsory school attendance. We divided the participants into those who dropped out of school during the 2010–2011 school year (cases) and those who still attended school or graduated and attained at least a basic qualification during or at the end of the 2010–2011 school year (controls). Cases and controls being so similar at the start of the 2010–2011 school year was supposed to avoid selection bias.

### Recruitment

**Fig 1:**
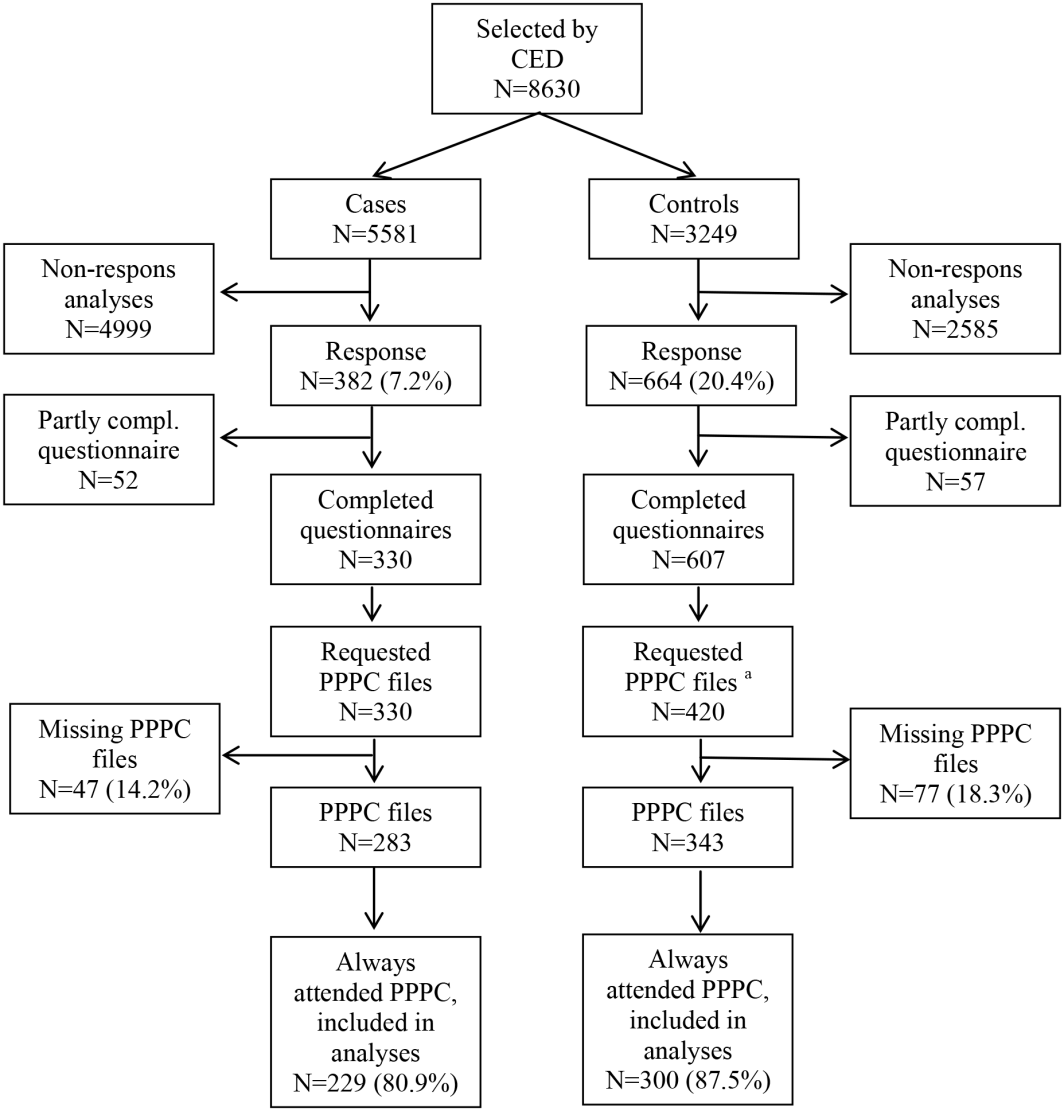
Flowchart of participant selection for the analyses. Abbreviations::CED compulsory educational department, PPPC preventive pediatric primary care, Compl. Completed. ^a^ randomly selected.

All eligible youngsters were sent a self-administered questionnaire with an informed consent form. In addition, we asked for the respondents’ permission to link the questionnaires to the PPPC files. A power analysis for the association between the experience of a chronic health condition in early life and the occurrence of early school leaving during adolescence indicated that 290 cases and 290 controls should be sufficient to yield an 80% power to detect an odds ratio of 1.75 at the conventional alpha level (5%) for an exposure prevalence of 0.2. More controls than cases returned and filled in the questionnaire. We included all cases with a completed questionnaire and took a random sample from the control group with a completed questionnaire. Subsequently we requested their PPPC file from the Public Health Service. We eventually were able to obtain the PPPC files of 283 cases and 343 controls out of the participants who completed the questionnaire, of which 229 cases (80.9%) and 300 controls (89.5%) had at least once visited the PPPC in every period of study (Fig 1).

### Ethics Statement

Approval for conducting this study was granted by the Medical Ethics Committee of Maastricht University (METC 11-4-099, decision 22-08-2011). More details can be found elsewhere [18].

### Measures

We used the standardized information from the PPPC files collected by the PPPC professionals, obstetricians or midwifes from birth onwards [21]. In addition, we used information from the questionnaires such as parental education and personality traits. All 71 potential relevant factors were selected in accordance with the literature and expert knowledge. In line with the routine health examinations in PPPC and the structure of the Dutch school system, we divided the variables according to four time periods (infancy 0–4 years, early childhood 4–8 years, middle childhood 8–12 years and adolescence 12–16 years) [22]. In every time period, the PPPC offers one or more non-mandatory health examinations. If the PPPC was attended at least once in every time period, and in the 0–4 time period at least twice (in the first year and at toddler age), we defined it as ‘always attended’ the PPPC.

#### School dropout

As described above, school dropouts (cases) were defined as 18 to 23 year olds who had dropped out of school during the 2010–2011 school year without attaining a basic qualification. Controls still attended school or had graduated and achieved at least a basic qualification during or at the end of the 2010–2011 school year.

#### Bio-psychosocial determinants

All determinants are enlisted and grouped in the tables (see the respective tables). Variables from the questionnaire are presented in italics.

The determinants are organized for each time period separately according to five domains: “health (physical and mental)”, “development”, “behavior, personality and lifestyle”, “participation” and “environment”. The time period 0–4 year additionally lists background sociodemographic variables and prenatal and perinatal variables.

#### Sociodemographic variables

The respondents’ own country of birth and that of their parents was used to categorize the young adults into Western (born in the Netherlands or originating from a country in Europe (not including Turkey), North America, Oceania, Indonesia or Japan) and non-Western ethnicity (originating from a country in Africa, South America, Asia or Turkey (not including Indonesia and Japan) [23]. Family composition was categorized as living with both parents or living in a one-parent family at the time that they attended primary school. Parental education is defined as the highest education completed, classified for both parents separately into low (no education, primary education, lower vocational education or pre-vocational theoretical program), intermediate (secondary vocational education, higher general secondary education or pre-university education) and high (higher professional education or university education) [24]. Socioeconomic background was based on the mean of the standardized scores for educational level of the father and the mother separately (each with originally eight ordinal categories) and four questions on material and social deprivation when at a primary school and at secondary school (each with four categories). The resulting variable was categorized into thirds using tertiles [25].

#### Prenatal and perinatal variables

Complications during pregnancy (hypertension, (pre)eclampsia, toxemia, HELLP syndrome or diabetes) and smoking, alcohol use, drug use or use of medication in pregnancy were dichotomized as “yes” if they were present and “no” or missing if they were not present or unknown. The Apgar score (range 1 tot 10) was measured after 1 and after 5 minutes. Preterm (gestational age of less than 37 weeks), microcephaly (head circumference less than the 3 percentile) and small for gestational age, defined as having a birth weight below -2 standard deviation scores [26] were dichotomized in “yes” in case of presence and “no” or “missing” if not present.

#### Health

Descriptions of physical and mental symptoms and illnesses and congenital abnormalities (with medical consequences) were categorized as “yes” in case of problems and “no” or “missing”. Physical illness excluded influenza, common colds and childhood diseases, such as varicella. Learning disorders, such as dyslexia or dyscalculia, attention problems, enuresis or encopresis, hospital admissions or surgery, teenage pregnancy and having experienced an accident (with a head injury, skin burns, intoxication or fracture) were dichotomized as a “yes” in case they were present and a “no” or “missing” if they were not present or unknown. Hospital admissions or surgery was chosen as a proxy for a (diagnosed) disease and included long-term hospitalizations and major surgery, but also and especially day-care admissions such as insertion of transtympanic tubes at a middle ear effusion. An insufficient visual acuity or hearing test yes/no is determined according to the applicable guidelines [21].

#### Development

We used the reference data for length and weight from the 1997 Dutch Growth Study [27]. Age of menarche is measured in years. Insufficient motor skills [28], language and speech problems and learning difficulties, including grade retention, were categorized as a “yes” in case any problems were present and as a “no” or “missing” if not present. Basic secondary education was classified into low (vocational pathways of a preparatory vocational secondary education), intermediate (theoretical learning pathway of a preparatory vocational secondary education) and high (senior general secondary education or pre-university education) [29].

#### Behavior, lifestyle and personality

Behavior problems were categorized in having internalizing or externalizing behavior (yes/no) according to the Child Behavior Checklist [30]. Lack of motivation, eating and sleeping difficulties and smoking, alcohol use and drug use were dichotomized as a “yes” if present and as a “no” or “missing” if they were not present. Rebelliousness, defined as “wanting or feeling compelled to do something contrary to that required by some external agency” was measured with 18 items from the Social Reactivity Scale [31]. The mean score was computed (0 = low and 2 = high rebelliousness) (Cronbach’s α = 0.66). Mastery, defined as “control over life and circumstances”, was measured by computing the mean of the seven items of the Pearlin and School scale (0 = low and 5 = high mastery) (Cronbach’s α = 0.83) [32]. General self-efficacy, thinking that you are able to perform certain behaviors, was measured by computing the mean on the 16 items of Sheffer’s general self-efficacy scale (0 = low and 5 = high self-efficacy) (Cronbach’s α = 0.89) [33]. For both mastery and general self-efficacy, the respondents were asked to think of the time when they were 16. Social inadequacy was measured with computing the mean on the 15 items of the Dutch Personality Questionnaire (0 = low inadequacy and 2 = high inadequacy) (Cronbach’s α = 0.87) [34]. Neuroticism and extraversion were each measured with 12 items from the Eysenck questionnaire [35]. Mean scores were computed ranging from 0 = low to 1 = high (Cronbach’s α = 0.84 for both neuroticism and extraversion).

#### Participation

Descriptions of participation in sports (including swimming lessons), having friends (1 or none/ 2 or more) and having a part-time job outside of school were categorized as “yes” in case of non-participation or not having friends or a part-time job and “no” or “missing” otherwise. School absenteeism (yes/no) was defined as truancy or absenteeism due to sickness.

#### Environment

Age of parents when the respondent was born was categorized into “younger than 25 years” and “25 years or older”. Birth order is defined as firstborn (1), second born (2), third born (3) etc. Family expansion (yes/no) is defined as the birth of a sibling (in every time period) or the moving in of a stepparent or a stepbrother or sister (in the time periods 4–8, 8–12 and 12–16 years). A bad relationship between parents, parental health problems, health problems of siblings, disturbed parent-child interaction and parenting problems were dichotomized as a “yes” in case of problems and as a “no” or “missing” otherwise. In case of suspected or identified child abuse, being bullied or being placed on a rebound facility (a facility in or outside school that provide temporary accommodation for pupils with behavioral problems) we categorized as “yes”, if not as “no” or “missing”. Descriptions of stressful life events were categorized as “yes” if present and “no” or “missing” if none were present. For the number of stressful life events, the events were summed.

### Statistical analyses

Descriptive statistics, including frequencies, were calculated for all variables. Differences in participation of the PPPC examinations between cases and controls were determined using Chi^2^ tests. To examine the association between relevant demographic variables (i.e. ethnic background) and PPPC attendance (always versus not always), we conducted logistic regression analyses for each variable separately. Subsequently, we examined the association between all variables and school dropout. Only participants who attended the PPPC at least once in every time period were included into the analyses.

First, we conducted univariate logistic regression analyses for each variable separately with school dropout as the dependent variable. To cross-validate our findings, we divided the group randomly into an exploratory and a confirmatory half. Variables were selected for further analysis if the odds ratios (OR) in both halves pointed in the same direction and at least the OR in the total group was significant (p<0.05) (because the power calculation was based on the total group).

Second, with the selected variables (bold variables in the respective tables), we conducted multivariable (stepwise) analyses beginning with the variables from the 0–4 period. Variables were selected for further analysis if the OR in both the exploratory and the confirmatory half pointed in the same direction and at least the OR in the total group was significant (p<0.05) in both the forward and backward stepwise analysis. As we aimed to identify risk factors as early in life as possible and therefore needed to avoid over-adjustment, we forced the selected variables from the 0–4 period into the next analyses in which the variables pertaining to the 4–8 period were added to a backward and forward stepwise regression analysis. Again, only if the OR in both halves pointed in the same direction and the OR in the total group was significant in the forward and stepwise regression analysis, variables were taken into the next age period. Subsequently, the selected variables from the 0–8 period were forced into the next analyses and variables from the 8–12 period were added in a backward and forward stepwise regression. Then, the selected variables from the 0–12 period were forced into the next analyses and variables from the 12–16 period were added in a backward and forward stepwise regression. This resulted in a final model with variables from the 0–16 period. Both the comparison of the forward and backward findings and the cross-validation in the two random halves of the sample were done to minimize capitalization on chance. We wanted to find determinants as early in life as possible and we therefore forced significant determinants in the early life phases into the models in which determinants from later life phases were tested. This simultaneously assures that determinants from later life phases are controlled for the possible confounding influence of the determinants from earlier life phases.

Finally, we conducted separate sensitivity analyses in which we looked at how ORs of the same variable differed in the results from the univariate and multivariable analyses.

## Results

Fig 1 shows that there were 8630 eligible young adults, of which 5581 were drop-outs and 3249 controls. Twelve percent of this group responded: 7.2 and 20.4 percent of cases and controls, respectively.

Non-participants were more likely to be men, dropouts (cases) and living in areas with cheaper houses (external zip code data [36]), and were slightly older than the participants (not tabulated). In the total group (including the eventual non-participants), men were more likely to have dropped out than women, indicating that the absence of a sex effect on dropout in the net sample is an underestimate.

As presented in Fig 1, our research sample in the current study consisted of 283 cases and 343 controls. Compared to controls, youngsters who dropped out of school more often missed health examinations from the PPPC in one or more time periods (n = 39 (11.8%) and n = 50 (17.9%), respectively (not tabulated)), resulting in an odds ratio of 1.62 (95% CI 1.03–2.55) (Table 1). Furthermore, youngsters with a non-Western background more often did not (always) attend the PPPC, as were youngster from one-parent families and youngsters living in areas with cheaper houses (OR 1.84; 95%CI 1.13–3.02) (not tabulated).

**Table 1:** Relevant demographic characteristics related to non-participation of the routine examinations of the Preventive Pediatric Primary Care (PPPC).

Variables	Missed ≥1 health examinations (n = 89)	
	Odds ratio	95% Confidence Interval
School dropout	1.62*	1.03–2.55
Sex; being a boy	1.09	0.69–1.71
Non-Western ethnicity	4.61*	2.47–8.61
One-parent family	3.13*	1.83–5.37
Education mother		
High education (ref)		
Intermediate education	0.64	0.31–1.33
Low education	1.29	0.68–2.44
Education father		
High education (ref)		
Intermediate education	0.96	0.47–1.85
Low educated father	0.94	0.51–1.73

*p<0.05

Tables 2 to 5 show the unadjusted odds of school dropout separately for variables pertaining to the four different time periods (age groups). Having parents aged under 25 years at time of birth (OR 2.00) was related to school dropout (Table 2). Furthermore, having a non-western background (OR 2.81) or living in a one-parent family (OR 1.85) increased the odds of dropout. In the 0–4 year period suffering from a physical illness (OR 1.47) and having an insufficient hearing test (OR 1.88) were related to dropout and so were not joining a nursery or preschool (OR 2.98) and a poor relationship between parents (OR 2.76). The experience of any additional stressful life event increased the odds of dropout (OR 1.32) and the birth of a sibling in the 0–4 year period decreased the odds (OR 0.56) (Table 2).

**Table 2:** Results from the univariate analyses of dropout (dependent variable) and bio-psychosocial determinants from the Preventive Pediatric Primary Care (PPPC) files and additional questionnaires in infancy (0–4 years). Only participants who always attended the PPPC are included into the analyses.

Variables in the time period 0–4 year from the PPPC files and questionnaire (italic)	Missing	Exploratory half	Confirmatory half	Total group	
		n = 266	n = 263	n = 529	95% Confidence
Number of adverse findings (n)	n (%)	Odds ratio	Odds ratio	Odds ratio	Interval
Sociodemographic variables					
Sex; boys (212)	0 (0.0)	1.20	0.91	1.04	0.73–1.48
**Non-western ethnicity (33)**	1 (0.2)	2.45**	3.41*	**2.81** *	**1.33–5.91**
***One-parent family* (69)**	0 (0.0)	3.48*	1.17	**1.85** *	**1.11–3.08**
*Socioeconomic background*	46 (8.7)				
High (169) (ref)					
Intermediate (112)		1.03	1.09	1.01	0.63–1.63
Low (202)		1.01	1.17	1.05	0.70–1.58
*Education father*	62 (11.7)				
High (127) (ref)					
Intermediate (140)		0.84	1.71	1.40	0.86–2.28
Low (200)		1.06	2.17*	1.11	0.71–1.74
*Education mother*	54 (10.2)				
High (93) (ref)					
Intermediate (172)		0.63	1.35	0.93	0.56–1.55
Low (210)		0.99	1.42	1.21	0.74–1.97
Prenatal and perinatal variables					
Pregnancy					
Smoking (104)	37 (7.0)	1.63	1.34	1.45**	0.94–2.23
Alcohol use (15)	35 (6.6)	5.27	0.36	0.91	0.32–2.61
Drug use (1)	35 (6.6)	-^d^	-	-	-
Medication use (191)	4 (0.8)	1.05	0.97	1.01	0.70–1.44
Complications (56)	1 (0.2)	0.74	0.68	0.71	0.40–1.25
APGAR after 1 minute^a^	200 (37.8)	0.95	0.99	0.99	0.82–1.19
APGAR after 5 minutes^a^	153 (28.9)	0.82	0.78	0.80	0.57–1.12
Premature birth (28)	0 (0.0)	1.47	0.94	1.14	0.53–2.45
SGA^b^ (24)	0 (0.0)	1.44	0.39	0.93	0.41–2.14
Microcephaly (12)	22 (4.2)	3.26	0.36	1.36	0.43–4.27
Health					
Congenital abnormality(6)	0 (0.0)	0.81	1.43	1.31	0.26–6.57
Perinatal asphyxia (18)	3 (0.6)	1.70	0.30	0.66	0.24–1.77
**Physical illness (312)**	0 (0.0)	1.54**	1.48	**1.47** *	**1.03–2.09**
Somatic complaints (289)	0 (0.0)	0.77	0.87	0.83	0.39–1.17
Enuresis/encopresis (183)	6 (1.1)	0.88	1.30	1.06	0.74–1.52
Mental illness (0)	0 (0.0)	-	-	-	-
Mental complaints (2)	0 (0.0)	-	-	-	-
Had an accident (34)	0(0.0)	1.59	0.63	1.04	0.52–2.09
Hospital admission or surgery (229)	0(0.0)	1.17	1.41	1.26	0.88–1.82
Insufficient visual acuity (94)	2 (0.4)	0.76	1.22	1.02	0.65–1.60
**Insufficient hearing test (74)**	0 (0.0)	1.61	2.29*	**1.88** *	**1.15–3.09**
Development					
Length as an infant	4 (0.8)				
P3-P97 (506) (ref)					
<P3 (9)		2.53	0.72	1.07	0.28–4.01
>p97 (5)		1.26	2.86	2.00	0.33–12.06
Diverging curve (5)		-	0.72	2.00	0.33–12.06
Weight as an infant	4 (0.8)				
P3-P97 (479) (ref)					
<P3 (15)		4.96	1.42	2.81**	0.95–8.36
>P97 (12)		-	0.47	4.22*	1.13–15.79
Diverging curve (19)		1.70	0.85	1.27	0.51–3.17
Length as a toddler	6 (1.1)				
P3-P97 (500) (ref)					
<P3 (7)		3.78	-	0.96	0.21–4.35
>P97 (9)		0.42	-	0.16**	0.20–1.29
Diverging curve (7)		1.26	0.33	0.51	0.10–2.67
Weight as a toddler	15 (2.8)				
P3-P97 (434) (ref)					
<P3 (35)		1.20	0.82	1.04	0.52–2.08
>P97 (32)		0.94	1.81	1.39	0.68–2.84
Diverging curve (13)		1.76	0.30	0.87	0.28–2.69
Insufficient motor skills (46)	0 (0.0)	0.47	0.64	0.55**	0.28–1.05
Language and speech problem (117)	3 (0.6)	1.60	0.77	1.13	0.75–1.7
Behavior, lifestyle and personality					
Externalizing behavior (257)	0 (0.0)	1.25	1.05	1.12	0.80–1.58
Internalizing behavior (129)	0 (0.0)	1.44	0.88	1.14	0.77–1.70
Eating difficulties (67)	0 (0.0)	1.28	1.17	1.23	0.74–2.06
Sleeping difficulties (111)	1 (0.2)	1.26	1.08	1.15	0.76–1.76
Participation					
**No nursery or preschool (16)**	0 (0.0)	7.75**	1.80	**2.98** *	**1.02–8.69**
Environment					
**Age parents aged <25 years (51)**	0 (0.0)	2.67*	1.72	**2.00** *	**1.11–3.60**
Family composition					
One child (40)	0 (0.0)	3.79*	0.54	1.34	0.70–2.55
≥ 4 Children (64)	0 (0.0)	0.82	1.60	1.10	0.65–1.86
Birth order^a^	0 (0.0)	1.07	1.29	1.17**	0.97–1.41
**Family expansion (232)**	0 (0.0)	0.47*	0.68	**0.56** *	**0.39–0.79**
Health problems of siblings (32)	0 (0.0)	0.99	0.55	0.77	0.37–1.62
Suspected or identified child abuse (2)	0 (0.0)	-	-	1.31	0.08–21.08
Experienced SLE^c^ (77)	1 (0.2)	0.62	1.12	1.59**	0.98–2.59
**Number of SLE** ^a^ ^,^ ^c^	0 (0.0)	1.34**	1.30**	**1.32** *	**1.07–1.62**
**Bad relationship between parents (27)**	0 (0.0)	1.78	4.21*	**2.76** *	**1.22–6.26**
Parental health problems (156)	3 (0.6)	0.99	0.99	0.98	0.67–1.43
Deceased parent (13)	0 (0.0)	1.04*	0.46	1.55	0.51–4.66
Disturbed parent child interaction (18)	0 (0.0)	0.40	0.59	0.49	0.17–1.40
Parenting problems (20)	0 (0.0)	0.52	2.18	1.08	0.44–2.64

*P<0.05,

** p<0.10,

^a^Continuous variable,

^b^ SGA = small for gestational age,

^c^ SLE = stressful life events.

^d—^ = not analyzed (n = 0 or only cases).

Variables from the questionnaire are in *italic*, variables included in the multivariable logistic regression analyses are in **bold**.

**Table 3:** Results from the univariate analyses of dropout (dependent variable) and biopsychosocial determinants from the Preventive Pediatric Primary Care (PPPC) files in early childhood (4–8 years). Only participants who always attended the PPPC are included into the analyses.

Variables in the time period 4–8 year from the PPPC files	Missing	Exploratory half	Confirmatory half	Total group	
		n = 266	n = 263	n = 529	95% Confidence
Number of adverse findings (n)	n (%)	Odds ratio	Odds ratio	Odds ratio	Interval
Health					
Physical illness (140)	1 (0.2)	1.23	0.99	1.09	0.74–1.61
Somatic complaints (84)	0 (0.0)	1.07	1.61	1.30	0.82–2.08
**Enuresis/encopresis (64)**	0 (0.0)	1.78	1.92**	**1.81** *	**1.07–3.07**
Mental illness (5)	0 (0.0)	1.24	-^c^	0.87	0.15–5.26
Mental complaints (23)	0 (0.0)	0.99	3.79*	2.11**	0.90–4.95
Attention problems (62)	0 (0.0)	1.28	2.48*	1.69**	0.99–2.89
Learning disability (9)	0 (0.0)	1.24	2.15	1.65	0.44–6.22
Had an accident (28)	0 (0.0)	2.61**	0.88	1.55	0.72–3.32
**Hospital admission or surgery (47)**	0 (0.0)	0.29*	0.38**	**0.33** *	**0.16–0.67**
Insufficient visual acuity (114)	0 (0.0)	1.30	1.03	1.18	0.78–1.79
Insufficient hearing test (99)	0 (0.0)	0.96	1.19	1.06	0.68–1.65
Development					
Length	1 (0.2)				
P3-P97 (503) (ref)					
<P3 (10)		6.40**	0.45	1.95	0.54–6.98
>P97 (11)		1.28	-	0.49	0.13–1.86
Diverging curve (4)		-	0.67	0.43	0.05–4.18
Weight	1 (0.2)				
P3-P97 (432) (ref)					
<P3 (45)		1.40	1.74	1.54	0.83–2.85
>P97 (50)		1.16	0.91	0.98	0.54–1.77
Diverging curve (1)		-	-	-	-
Insufficient motor skills (76)	1 (0.2)	1.53	0.54	1.01	0.62–1.65
Language and speech problems (112)	1 (0.2)	1.87*	0.75	1.30	0.85–1.97
**Learning difficulties (62)**	0 (0.0)	2.08**	1.87	**1.97** *	**1.15–3.36**
Behavior, lifestyle and personality					
Externalizing behavior (126)	1 (0.2)	0.73	1.13	0.93	0.62–1.40
Internalizing behavior (163)	1 (0.2)	1.46	1.09	1.26	0.87–1.82
Eating difficulties (36)	0 (0.0)	0.84	1.95	1.19	0.60–2.34
Sleeping difficulties (34)	0 (0.0)	1.13	0.88	1.04	0.52–2.09
Participation					
**Not attending sports (21)**	0 (0.0)	3.46**	3.45**	**3.44** *	**1.31–9.00**
School absenteeism (3)	0 (0.0)	2.61**	0.88	2.63	0.24–29.14
Not having friends (25)	2 (0.4)	0.97	0.69	0.87	0.38–1.97
Environment					
Being bullied (17)	3 (0.6)	0.62	1.71	1.17	0.44–3.08
Family expansion (54)	0 (0.0)	0.95	1.02	0.97	0.55–1.71
Suspected or identified child abuse (8)	0 (0.0)	2.50	2.15	2.21	0.52–9.34
Experienced SLE^b^ (161)	0 (0.0)	0.71	1.29	0.94	0.65–1.37
Number of SLE^a^ ^,^ ^b^	0 (0.0)	0.97	1.24	1.08	0.86–1.37
**Bad relationship between parents (32)**	0 (0.0)	1.59	3.79*	**2.30** *	**1.10–4.80**
Parental health problems (23)	2 (0.4)	2.60	1.18	1.75	0.75–4.06
Disturbed parent child interaction (25)	0 (0.0)	2.55	1.89	2.03**	0.90–4.61
Parenting problems (18)	0 (0.0)	0.60	1.43	0.83	0.32–2.17

*P<0.05,

** p<0.10,

^a^ Continuous variable,

^b^ SLE = stressful life events,

^c—^ = not analyzed (n = 0 or only cases).

Variables included in the multivariable logistic regression analyses are in **bold**.

**Table 4:** Results from the univariate analyses of dropout (dependent variable) and biopsychosocial determinants from the Preventive Pediatric Primary Care (PPPC) files in middle childhood (8–12 years). Only participants who always attended the PPPC are included into the analyses.

Variables in the time period 8–12 year from the PPPC files	Missing	Exploratory half	Confirmatory half	Total group	
		n = 266	n = 263	n = 529	95% Confidence
Number of adverse findings (n)	n (%)	Odds ratio	Odds ratio	Odds ratio	Interval
Health					
Physical illness (53)	3 (0.6)	1.58	1.02	1.29	0.73–2.28
**Somatic complaints (74)**	3 (0.6)	1.34	2.30	**1.76** *	**1.07–2.89**
Enuresis/encopresis (20)	4 (0.8)	0.49	1.23	0.87	0.35–2.17
Mental illness (17)	1 (0.2)	0.45	0.70	0.53	0.19–1.54
Mental complaints (30)	1 (0.2)	2.87**	1.06	1.76	0.84–3.71
**Attention problems (43)**	6 (1.1)	3.15*	1.21	**1.96** *	**1.04–3.69**
Learning disability (16)	5 (0.9)	1.49	0.72	1.34	0.50–3.63
Had an accident (14)	2 (0.4)	1.56	2.14	1.76	0.60–5.15
Hospital admission or surgery (7)	1 (0.2)	3.75	0.70	1.75	0.39–7.92
Insufficient visual acuity (131)	5 (0.9)	1.33	1.03	1.19	0.80–1.77
**Insufficient hearing test (27)**	6 (1.1)	1.07	5.54*	**2.32** *	**1.04–5.16**
Development					
Length	0 (0.0)				
P3-P97 (504) (ref)					
<P3 (6)		1.88	-^c^	1.29	0.26–6.46
>P97 (16)		1.00	0.23	0.59	0.20–1.71
Diverging curve (3)		-^c^	0.68	0.65	0.06–7.16
Weight	0 (0.0)				
P3-P97 (424) (ref)					
<P3 (26)		0.84	1.14	0.95	0.43–2.11
>P97 (78)		0.89	0.95	0.90	0.55–1.47
Diverging curve (1)		-	-		
**Menarche age** ^a^ **, girls only (219)**	98 (30.9)	0.53*	0.97	**0.73** *	**0.55–0.98**
Insufficient motor skills (30)	6 (1.1)	1.89	0.47	1.35	0.65–2.82
Language and speech problems (36)	7 (1.3)	1.26	0.89	1.05	0.53–2.08
Learning difficulties (48)	0 (0.0)	1.03	1.74	1.35	0.74–2.44
Behavior, lifestyle and personality					
**Externalizing behavior (64)**	6 (1.1)	4.48*	1.74	**2.85** *	**1.65–4.94**
Internalizing behavior (87)	6 (1.1)	1.52	1.54	1.51**	0.95–2.40
Eating difficulties (14)	6 (1.1)	4.54**	0.95	2.42	0.80–7.33
Sleeping difficulties (35)	6 (1.1)	1.44	2.35**	1.83**	0.91–3.65
Participation					
Not attending sports (143)	15 (2.8)	1.15	1.46	1.26	0.86–1.86
School absenteeism (5)	3 (0.6)	-	-	-	-
Not having friends (21)	4 (0.8)	2.61	1.15	1.81	0.75–4.37
Environment					
Being bullied (45)	6 (1.1)	1.76	1.04	1.27	0.69–2.35
**Family expansion (18)**	1 (0.2)	9.06*	2.17	**3.54** *	**1.24–10.08**
Suspected or identified child abuse (8)	2 (0.4)	-	2.14	3.98**	0.80–19.92
Experienced SLE^b^(130)	1 (0.2)	3.07*	0.92	1.49**	1.00–2.21
**Number of SLE** ^a^ ^,^ ^b^	1 (0.2)	1.88*	0.91	**1.32** *	**1.02–1.70**
**Bad relationship between parents (53)**	2 (0.4)	4.70*	1.37	**2.14** *	**1.20–3.83**
Parental health problems (25)	497 (94.0)	1.67	1.25	1.13	0.21–6.14
Disturbed parent child interaction (18)	7 (1.3)	0.20	2.61	1.07	0.41–2.75
Parenting problems (10)	6 (1.1)	0.82	0.95	0.87	0.24–3.13

*P<0.05,

** p<0.10,

^a^ Continuous variable,

^b^ SLE = stressful life events,

^c—^ = not analyzed (n = 0 or only cases or controls).

Variables included in the multivariable logistic regression analyses are in **bold**.

**Table 5:** Results from the univariate analyses of dropout (dependent variable) and biopsychosocial determinants from the Preventive Pediatric Primary Care (PPPC) files and additional questionnaires in adolescence (12–16 years). Only participants who always attended the PPPC are included into the analyses.

Variables in the time period 12–16 year from the PPPC files and questionnaire (*italic*)	Missing	Exploratory half	Confirmatory half	Total group	
		n = 266	n = 263	n = 529	95% Confidence
Number of adverse findings (n)	n (%)	Odds ratio	Odds ratio	Odds ratio	Interval
Health					
Physical illness (76)	2 (0.4)	1.73	1.25	1.45	0.89–2.36
Somatic complaints (265)	2 (0.4)	1.59**	0.77	1.09	0.77–1.54
Mental illness (25)	1 (0.2)	0.85	1.43	1.03	0.46–2.31
**Mental complaints (27)**	1 (0.2)	2.56	1.66	**2.32** *	**1.04–5.16**
Attention problems (40)	5 (0.9)	2.72**	0.98	1.52	0.80–2.90
Learning disability (25)	0 (0.0)	2.05	1.44	1.71	0.76–3.84
Had an accident (23)	4 (0.8)	1.57	1.96	1.75	0.75–4.07
Hospital admission or surgery (23)	1 (0.2)	1.24	1.66	1.45	0.63–3.34
*Teenage pregnancy* (5)	46 (8.7)	-^c^	-	-	-
Development					
Length	3 (0.6)				
P3-P97 (503) (ref)					
<P3 (5)		1.26	0.69	0.87	0.14–5.26
>P97 (14)		0.94	0.55	0.73	0.24–2.20
Diverging curve (4)		-	0.69	1.31	0.18–9.36
Weight	3 (0.6)				
P3-P97 (418) (ref)					
<P3 (32)		1.33	0.80	1.07	0.52–2.21
>P97 (75)		1.41	1.18	1.27	0.78–2.08
Diverging curve (1)		-	-	-	-
Insufficient motor skills (5)	3 (0.6)	3.82	-	1.99	0.33–12.00
Language and speech problems (10)	2 (0.4)	6.42**	0.47	1.99	0.56–7.15
*Basic secondary education*	46 (8.7)				
High (116) (ref)					
Intermediate (225)		1.38	1.09	1.26	0.80–1.99
Low (142)		1.42	1.85	1.62**	0.99–2.67
**Learning difficulties (50)**	0 (0.0)	2.70*	2.07**	**2.32** *	**1.27–4.22**
Behavior, lifestyle and personality					
Externalizing behavior (109)	1 (0.2)	0.90	2.39*	1.50**	0.98–2.29
Internalizing behavior (154)	1 (0.2)	1.16	1.04	1.09	0.74–1.58
Lack of motivation (19)	20 (3.8)	0.90	4.76*	2.34**	0.91–6.06
Eating difficulties (43)	2 (0.4)	5.35*	0.50	2.14*	1.13–4.04
Sleeping difficulties (94)	2 (0.4)	1.29	1.54	1.47**	0.94–2.29
**Smoking (22)**	12 (2.3)	5.89*	4.08*	**4.77** *	**1.73–13.14**
Alcohol use (197)	18 (3.4)	1.47	1.31	1.36**	0.95–1.95
**Drug use (11)**	17 (3.2)	4.96	7.63**	**6.13** *	**1.31–28.68**
***Neuroticism*** ^a^	0 (0.0)	1.46	6.31	**2.97** *	**1.51–5.82**
***Extraversion*** ^*a*^	0 (0.0)	0.68	0.32*	**0.49** *	**0.25–0.96**
***Social- inadequacy*** ^a^	0 (0.0)	1.41	2.01*	**1.63** *	**1.13–2.34**
***Self-efficacy*** ^a^	0 (0.0)	0.61*	0.35*	**0.47** *	**0.35–0.65**
***Mastery*** ^a^	0 (0.0)	0.56*	0.60*	**0.58** *	**0.44–0.75**
***Rebelliousness*** ^a^	46 (8.7)	2.52*	2.46**	**2.45** *	**1.20–4.99**
Participation					
**Not attending sports (122)**	7 (1.3)	1.88*	1.53	**1.69** *	**1.13–2.55**
**School absenteeism (36)**	1 (0.2)	19.33*	5.13*	**7.36** *	**3.01–18.01**
Not having friends (44)	1 (0.2)	0.62	0.38**	0.52**	0.27–1.02
Part-time employment (29)	1 (0.2)	3.68*	1.07	1.92**	0.90–4.10
Environment					
Being bullied (72)	1 (0.2)	2.16*	0.64	1.28	0.78–2.10
Family expansion (16)	11 (2.1)	6.37**	1.45	2.24	0.80–6.27
Suspected or identified child abuse (8)	1 (0.2)	1.23	7.36**	4.00**	0.80–19.98
**Experienced SLE** ^b^ **(142)**	1 (0.2)	2.07*	1.26	**1.56** *	**1.06–2.30**
**Number of SLE** ^a^ ^,^ ^b^	1 (0.2)	1.99*	1.22	**1.43** *	**1.16–1.76**
**Bad relationship between parents (73)**	1 (0.2)	3.70*	1.52	**2.07** *	**1.25–3.43**
Parental health problems (24)	498 (94.1)	-	0.75	2.96	0.48–18.34
**Disturbed parent child interaction (32)**	3 (0.6)	3.01	1.77	**2.29** *	**1.10–4.79**
Parenting problems (19)	6 (1.1)	1.60	0.94	1.49	0.60–3.74
***Placed on a rebound facility* (29)**	46 (8.7)	3.40*	2.52	**2.90** *	**1.29–6.51**

*P<0.05,

** p<0.10,

^a^ Continuous variable,

^b^ SLE = stressful life events,

^c—^ = not analyzed (n = 0 or only cases).

Variables from the questionnaire are in *italic*, variables included in the multivariable logistic regression analyses are in **bold**.

Table 3 shows that enuresis or encopresis (OR 1.81), learning difficulties (OR 1.97) and not attending sports were risk factor for later school dropout in the 4–8 time period (OR 3.44) and so was a poor relationship between parents (OR 2.30). A hospital admission or surgery in this time period decreased the odds (OR 0.33).

Variables of the 8–12 time period are shown in Table 4. Family expansion (OR 3.54), somatic complaints (1.76), attention problems (OR 1.96), externalizing behavior (OR 2.85) and having an insufficient hearing test (OR 2.32) were related to dropout and so was a poor relationship between parents (OR 2.14). For girls, age of menarche played a role, as for each year between the age of 10 and 15 years the menarche delayed, the odds decreased with 0.73. The experience of any additional stressful life event in the 8 to 12 time period increased the odds of dropout with 1.32.

Table 5 shows that in the 12–16 year period, variables significantly related to dropout are spread across all domains with the emphasis on behavior, lifestyle and personality (smoking (OR 4.77), drug use (OR 6.13), neuroticism (OR 2.97), extraversion (OR 0.49), social inadequacy (OR 1.63), self-efficacy (OR 0.47), mastery (OR 0.58) and rebelliousness (OR 2.45)). Adolescents aged 12 to 16 years, had higher odds of dropout when they were placed on a rebound facility (OR 2.90) or when their relation with their parents was disturbed (OR 2.29). However, a poor relationship between their parents also increased the odds (OR 2.07). Furthermore mental complaints, learning difficulties, not attending sports and school absenteeism were related to school dropout (OR 2.32, OR 2.32, OR 1.69 and OR 7.36 respectively). The experience of stressful life events increased the odds of dropout (OR 1.56). Moreover, the experience of any additional stressful life event increased the odds with 1.43. Teenage pregnancy occurred in five girls who always attended the PPPC, but an additional 11 girls reported a teenage pregnancy in the questionnaire (not tabulated). These were all cases.

The significant variables (in bold in Tables 2 to 5) were subsequently introduced in the stepwise regression analyses. Remaining variables from an earlier period were forced (not allowed to be excluded from the model) into the next stepwise model with candidate variables from later periods. Table 6 presents the final model of these age-attentive stepwise regression analyses. Having a non-Western background (OR 2.54, 95% CI 1.10–5.89) and an insufficient hearing test in the 0–4 time period (OR 1.75, 95% CI 0.98–3.10) increased the odds of dropout. The birth of a sibling was protective in this age period (OR 0.63, 95% CI 0.43–0.93). Learning difficulties (OR 1.74, 95% CI 0.95–3.18) and not attending sports (OR 2.88, 95% CI 0.99–8.42) were risk factors in the 4–8 time period, whereas hospitalization or surgery (OR 0.29 95% CI 0.13–0.63) in this period decreased the odds of dropout. In the 8–12 time period, externalizing behavior (OR 2.81 95% CI 1.53–5.14) was a risk factor and so was school absenteeism in the 12–16 year period (OR 5.62, 95% CI 2.18–14.52). Higher self-efficacy (scale 0–5) decreased the odds of dropout with 0.53 for every one unit increase on the self-efficacy scale (OR 0.53, 95% CI 0.38–0.74).

**Table 6:** Determinants in infancy, early and middle childhood, and adolescence predicting school drop-out in young adulthood (18–23 years). The final model.

Variables from the PPPC^a^ files and questionnaires (*italic*) n = 529		95% Confidence
	Odds ratio	Interval
**Age 0–4 years**		
Sociodemographic		
Non-Western ethnicity	2.54*	1.10–5.89
Health		
Insufficient hearing test 0–4	1.75**	0.98–3.10
Environment		
Family expansion 0–4	0.63*	0.43–0.93
**Age 4–8 years**		
Health		
Hospitalization or surgery 4–8	0.29*	0.13–0.63
Development		
Learning difficulties 4–8	1.74**	0.95–3.18
Participation		
Not attending sports 4–8	2.88**	0.99–8.42
**Age 8–12 years**		
Behavior, lifestyle and personality		
Externalizing behavior 8–12	2.81*	1.53–5.14
**Age 12–16 years**		
Participation		
School absenteeism 12–16	5.62*	2.18–14.52
Behavior, lifestyle and personality		
*Self-efficacy* ^*b*^ 12–16	0.53*	0.38–0.74

*p<0.05,

**p<0.10,

^a^ PPPC = Preventive Pediatric Primary Care,

^b^ continuous variable

Variables from the questionnaire are in italic

In the sensitivity analysis, we found some evidence for mediational pathways towards school dropout. The most major decreases in ORs, when controlling for a possible mediating variable (from a later life phase), occurred first for insufficient hearing at 0–4 years which was no longer significantly related to school dropout in the final model (Table 6) when controlled for learning difficulties in the 4–8 time period (not tabulated). Second, although non-western ethnicity was still significant in the final model, it decreased in association particularly by increasing non-attendance at sports in the 4–8 time period. Third, learning difficulties in the 4–8 time period were related to dropout partly because of increasing risks of externalizing behavior in the 8–12 time period. In another sensitivity analysis, we found no difference in associations, when socioeconomic status and sex were additionally controlled for in the final model: both socioeconomic status and sex were not significantly related to school dropout, nor was the pattern of associations of the other variables with school dropout substantially different from the pattern reported in Table 6.

## Discussion

Using life course data from the PPPC files, collected in the everyday practice of the PPPC professionals and using an additional questionnaire, we identified risk factors and protective factors on the pathway to school dropout from birth onwards. Whereas most studies focus on single determinants, our multidimensional approach enabled us, in one study, to explore many candidate biopsychosocial determinants that might be related to school dropout in young adulthood. An age-attentive life-course approach in the modeling enabled us to identify these factors early in life and, hence, to provide tools for future interventions aimed at reducing school dropout at an early stage. Our findings supported the relevance (for school dropout) of having a non-Western background, an insufficient hearing test and the birth of a sibling (protective) in the 0–4 age period, learning difficulties, not attending sports and hospitalization or having surgery (protective) in the 4–8 age period, externalizing behavior in the 8–12 age period and school absenteeism and self-efficacy (protective) in the 12–16 age period.

Some of these determinants are not or less amenable to preventive interventions than others. However, awareness of how these determinants increase the risk of later dropout can contribute to preventive interventions for children with heightened odds of school dropout. Furthermore, the biopsychosocial domains and determinants cumulate and interact over time. Socio demographic determinants like having a non-Western ethnicity is a proxy for culturally different practices or behaviors or circumstances which we have not separately measured, factors which may be more or less amenable for prevention. Besides, our findings in the sensitivity analysis that not attending sports at the age of 4–8 years decreased the odds of school dropout for having a non-Western ethnicity suggest a pathway to school dropout through non-participation. Additionally, children with a non-Western ethnicity more often missed one or more health examinations of the PPPC. Our finding that not participating in sports at the age of 4–8 years increases the odds of school dropout is in line with other findings that participation in organized leisure activities in childhood is associated with lower dropout rates [37]. A better response to no-show of non-Western families at the PPPC and stimulating their participation in society including sports may thus contribute to lower dropout rates. Truancy, particularly when it is persistent or frequent, is a well-known risk for school dropout [8]. Furthermore, children who are frequently or persistently absent from school due to illness also tend to perform poorly in school and might therefore be at higher risk of school dropout [38]. In accordance with other studies, we found academic problems to be a risk factor for dropout even in the first grades of primary school (4–8 age period) [8,39]. The OR of learning difficulties in the 4–8 time period (OR 1.97; 95%CI 1.15–3.36) decreased to an OR of 1.83 (95% CI 1.06–3.16), when externalizing behavior in the 8–12 time period was added. This suggests a pathway to school dropout through externalizing behavior. Children with early externalizing problems are more likely to become rejected and socially excluded in adolescence which may heighten their risks of dropout [40, 38]. According to other studies, we found externalizing behavior in the age of 8 to 12 years associated to later school dropout [3,8].

Youngsters’ less likely dropping out of school when they had got a sibling in infancy is in line with studies reporting that children without brothers or sisters are at higher risk of dropping out [41]. Possibly, the birth of a sibling provides a larger social network and practice to function within a group. Furthermore, parental attention has to be shared which increases self-dependence, but can also give competition which makes children more defensible. In our study, self-efficacy, which stimulates the student’s drive and thus school attendance [40], is associated with less school dropout [11]. This suggests that increasing self-efficacy at an early age may help to prevent school dropout. Children with hearing problems at a young age (0–4 year) have higher odds of school dropout, however additional learning difficulties in the 4 to 8 time period or externalizing behavior at the age of 8–12 year decreased the odds of dropout for hearing problems, suggesting that the pathway to dropout for hearing problems in the 0–4 time period leads through learning problems at the age of 4–8 year and externalizing behavior at the age of 8–12 year. We further found more youngsters having surgery or being hospitalized at the age of 4–8 years in the control group. Most surgeries concerned day admissions for hearing loss due to middle ear effusion, which is usually detected at the PPPC health examinations. Dropouts more often missed one or more health examinations; it is therefore possible that hearing problems remained undetected and caused learning or behavior problems. Furthermore, parents of the controls may be more closely watching the health status of their children or perhaps being in the hospital in early childhood brings positive attention from teachers and peers in primary school. Since we are not sure of the explanation, further research is required. Although we did not include menarche age and teenage pregnancy in the multivariable regression analyses, because they are only relevant in girls, we should consider it risk factors for school dropout, in line with other studies [1,42]. Girls with an early menarche have higher rates of externalizing problems and school problems [43,44], which may explain our finding that a younger age at menarche is a risk factor for school dropout. Besides, early puberty is associated with teenage pregnancy, which in turn is a risk for dropout [45,46].

School dropout reduces the chance of finding a job [47] and low socioeconomic status is associated with poorer health [48]. Furthermore, parental socioeconomic status is a strong predictor of a child’s academic achievement [15]. Since school dropout is also a public health problem, PPPC might play a role in breaking this negative spiral by monitoring children with an increased dropout risk from birth onwards and empower their strengths. However one-parent families and families with a non-Western background, who often have a lower income, participate less in the health examinations of the PPPC and so do families living in areas with cheaper houses. Therefore, the effect of socioeconomic status in our study might be underestimated. Moreover, non-participation in the PPPC might increase the socioeconomic health differences and inequalities. Especially families suffering from multiple and complex problems are a hard-to-reach group [49]. To reach these families, an active approach is required in addition to a more easily accessible and approachable PPPC [50].

We used candidate determinants from the whole life-course and, acknowledging the biopsychosocial context, from various domains. Many determinants are collected in PPPC practice and interventions may, we hope, easily build upon this practice. Furthermore, our design and approach was highly systematic in trying to exclude selection bias and capitalization on chance and trying to particularly appreciate the influence of determinants from earlier life. Falsely removing the statistically significant early life determinants -due to overadjustment as a result of controlling for mediators- did not occur because these early phase determinants were forced into the model with candidate determinants from later phases (where mediational processes could take place). We admit that overadjustment could still have resulted in underestimated odds ratios in the multivariable analyses, but this was evaluated further as possible mediational processes in the sensitivity analyses, where the most major processes were described. The forced entry of the initially significant early life phase determinants also controls for their potential confounding influence on the associations of later life phase determinants and school dropout.

Our approach may still have led to an oversimplified representation of the pathways towards school dropout. Given the abundance of candidate determinants and the resulting difficulty of specifying direct acyclic graphs (DAGs)[51, 52] and the many specified pathways and mechanisms in such graphs, we could, however, not think of other more insightful statistical approaches of the inter-individual variation in pathways towards school dropout. Although our model is thus also too simple to exclude unmeasured mediator-outcome confounding, interactions between variables, and intermediate confounding [53], we still think it provides robust quantitative evidence on the major early life determinants of later school dropout. However, to get further insight into and understanding of the complex pathways, we also conducted interviews with participants and focus groups with parents, teachers and PPPC physicians that will help to further interpret and contextualize the results. These will also further explore the needs and requirements of all stakeholders to prevent school dropout. Our aim with both the current quantitative and the future qualitative information is to develop a personalized prediction model and ultimately an effective intervention that is able to prevent dropout at an early stage.

There are some further limitations that need to be addressed. First, this study has an exploratory character, but the systematic way of analyzing by comparing the forward and backward findings and the cross-validation in the two random halves of the sample minimizes capitalization on chance. The model certainly needs further validation in other samples, but also across various subgroups (e.g. according to sex, ethnic background, socioeconomic status etc.). Second, to avoid selection bias, the control group was highly similar to the cases at the start of the school year 2010–2011 (both were still attending school). The reported odds ratios thus only apply to an already initially disadvantaged population, as, at the age of 18 to 23, both cases and controls already should have attained their basic qualification. Simultaneously, this might imply that we have underestimated the associations compared to a study using all possible controls and cases. Although, for future studies, we recommend a longitudinal design in which all possible cases and controls, at any age, would be included, we think that our efforts in avoiding selection bias has provided valid information on determinants of school dropout. Further selection bias may have taken place, since only available PPPC files from children who previously completed a questionnaire and who visited the PPPC in every time-period are included into the analysis. We therefore compared participants and non-participants regarding their dropout status, age, sex and zip-code level socioeconomic status [36]. Since men, dropouts (cases) and youngsters living in areas with cheaper houses less often completed the questionnaire and cases, youngsters with a non-Western background, living in one-parent families or in areas with cheaper houses or having a lower educated mother more often missed one or more PPPC health examinations, the absence of a sex and socioeconomic status effect on dropout in the final sample is probably an underestimate. Third, information bias is also of relevance. Some determinants, for example child abuse, have a low prevalence in our study and may therefore not be identified as a risk factor despite its high ORs. Possibly, this is underreported in the PPPC files. Further research with a larger sample is needed. Furthermore, the data described in the PPPC files were not collected for the purpose of this study, so important information might be missing. In order to obviate this, an additional questionnaire was sent, in which we asked for personality traits thinking of the time when they were 16 years of age. However, recall bias still cannot be excluded. As the information in the PPPC files is collected from birth onwards, this information is not affected by recall bias. However, the data in the PPPC files have been compiled by many different PPPC professionals, each with their own interpretations and descriptions of certain situations, symptoms and life-course determinants. Because the PPPC professionals use this information in their daily practice, the ecological validity of our findings should, on the other hand, be high.

### Conclusion

To prevent school dropout, PPPC professionals should not wait until imminent dropout, but they should try to identify and tackle risk factors in childhood as early as possible. They can advise parents on understanding the long-term effects of hearing problems in infancy and the importance of participation in sports in early childhood, and support parents to strengthen protective factors such as giving a child positive attention and promoting self-efficacy. Furthermore, an important reduction of school dropout can also be attained by more actively approaching and helping parents and youngsters who withdraw from public health care. Further research with a larger sample is needed to validate our model across subgroups and to evaluate whether targeting the risk factors is truly effective in tackling school dropout. Future interviews and focus groups will help to further interpret and contextualize the results and to emphasize the wide individual differences in the context of school dropout.
